# Personalized Prediction of Acquired Resistance to EGFR-Targeted Inhibitors Using a Pathway-Based Machine Learning Approach

**DOI:** 10.3390/cancers11010045

**Published:** 2019-01-04

**Authors:** Young Rae Kim, Yong Wan Kim, Suh Eun Lee, Hye Won Yang, Sung Young Kim

**Affiliations:** 1 Department of Biochemistry, School of Medicine, Konkuk University, 120, Neungdong-ro, Gwangjin-gu, Seoul 05029, Korea; youngrae@gmail.com (Y.R.K.); yongwankim87@gmail.com (Y.W.K.); sephinlee@gmail.com (S.E.L.); 2 School of Medicine, Trinity Biomedical Sciences Institute, Trinity College Dublin, 152-160 Pearse Street, D02 R590 Dublin, Ireland; hyewonheidi@hotmail.com

**Keywords:** drug resistance, gefitinib, erlotinib, biostatistics, bioinformatics

## Abstract

Epidermal growth factor receptor (EGFR) inhibitors have benefitted cancer patients worldwide, but resistance inevitably develops over time, resulting in treatment failures. An accurate prediction model for acquired resistance (AR) to EGFR inhibitors is critical for early diagnosis and according intervention, but is not yet available due to personal variations and the complex mechanisms of AR. Here, we have developed a novel pipeline to build a meta-analysis-based, multivariate model for personalized pathways in AR to EGFR inhibitors, using sophisticated machine learning algorithms. Surprisingly, the model achieved excellent predictive performance, with a cross-study validation area under curve (AUC) of over 0.9, and generalization performance on independent cohorts of samples, with a perfect AUC score of 1. Furthermore, the model showed excellent transferability across different cancer cell lines and EGFR inhibitors, including gefitinib, erlotinib, afatinib, and cetuximab. In conclusion, our model achieved high predictive accuracy through robust cross study validation, and enabled individualized prediction on newly introduced data. We also discovered common pathway alteration signatures for AR to EGFR inhibitors, which can provide directions for other follow-up studies.

## 1. Introduction

Despite the initial benefits of EGFR inhibitors in cancer patients harboring EGFR mutations, the rapid development of acquired resistance (AR) is a major obstacle in clinical practice and often leads to therapeutic failure and disease recurrence. A broad range of mechanisms of AR to EGFR inhibitors have been proposed, from mutational to non-mutation-based mechanisms. However, the exact mechanisms still remain unclear due to the multifactorial natures of cancer and intracellular signaling networks. Inherent crosstalk and redundancy of signaling pathways introduces huge complexity [1,2]. Therefore, inhibiting a single signaling network via drugs may trigger other survival pathways and limit efficacy. These complex dynamics make it more difficult to understand the underlying causes of AR and predict potential EGFR inhibitor sensitivity.

With the recent growth of publically available genomic data, meta-analysis and computational modeling have emerged as key tools to overcome the limitations of insufficient statistical power in individual studies. Conventional meta-analysis methods are often univariate, performing statistical analysis on each feature independently. As conventional classification algorithms tend to overfit high-throughput datasets, also known as high dimension low sample size (HDLSS) datasets, analyses are practically infeasible, resulting in lower accuracy rates when the model is applied to blind data [3,4]. In recent years, regularized regression classifiers such as lasso and elastic net have emerged as more effective ways to perform feature selection and prediction in high dimensional data [4]. These methods modify the conventional ordinary least squares model, using a sparsity penalty that shrinks regression coefficients by imposing a constraint on their size. While this penalty function pushes some coefficients towards zero and introduces some bias, the decrease in variance can potentially improve predictive performance on new, unseen data. These techniques are more interpretable than alternative state-of-the-art algorithms such as support vector machines (SVM), artificial neural networks (ANN), and random forests, which are often considered to be black box models [5]. It is hard to interpret these alternative models, since their inner workings are incomprehensible. Model interpretability and parsimony are especially important in medical field, where numbers of predictors are much larger than sample sizes. In this aspect, regularized regression classifier is regarded as the most optimal model, since it has both more interpretability and similar or superior predicting performance compared with the alternative algorithms. Another possible strategy that reduces model complexity and increases interpretability is the pathway-based approach, which has the potential to better reflect the heterogeneous nature of cancer pathophysiology, compared to classical single gene- or molecule-based methods.

Early detection of acquired EGFR inhibitors resistance is critical, and can help physicians establish a treatment plan by predicting the outcome of a disease. However, previous prediction models are often only applicable to specific types of EGFR tyrosine kinase inhibitors (TKIs), provide insufficient sensitivity or specificity for other types of EGFR inhibitors, and fail to detect generalized predictors.

In this study, using a sophisticated penalized machine learning technique, we built a meta-analysis-based, multivariate model for personalized pathways in acquired EGFR inhibitor resistance. This resulted in a more interpretable and robust model with high generalized predictive performance throughout various EGFR inhibitors and cancer types.

## 2. Results

**Figure 1:**
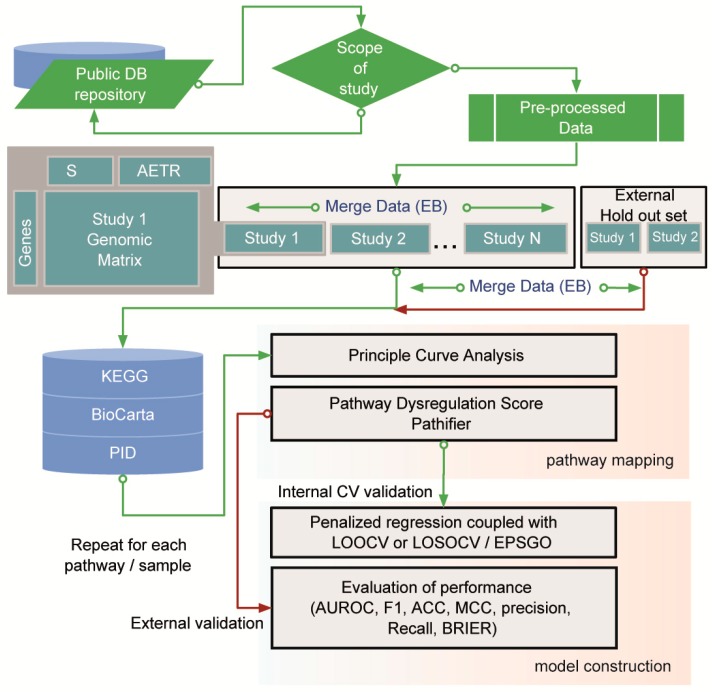
Pipeline for performing a meta-analysis-derived, multivariate model for personalized pathways in acquired epidermal growth factor inhibitor tyrosine kinase inhibitor (EGFR TKI) resistance (AETR). The pipeline consists of three main parts: cross study normalization, pathway mapping, and prediction model construction. The study cohort was preprocessed and categorized into an internal training/validation study set (N) and an external validation study set (M). For cross-study normalization, an empirical Bayes (EB) method was used. Pathway mapping for each individual sample was conducted using a Pathifier algorithm and public pathway databases (KEGG, BioCarta, and PID). The regularized regression model was built using elastic net. The optimal values of the hyper-parameters α and λ for elastic net regression were obtained from robust cross validation (leave-one-study-out cross validation (LOSOCV) or leave-one-out cross validation (LOOCV)) with Efficient Parameter Selection via Global Optimization (EPSGO) algorithm. S, sensitive.

To build a robust and generalized prediction model based on individualized pathway information, we developed a novel pipeline that integrates meta-analysis-based regularized regression with pathway-level measurement of abnormality (Figure 1). A total of 8 studies, all of which followed the strict AR criteria mentioned in the methods section, were used for model building. The study cohort was very heterogeneous in terms of the types of EGFR inhibitors, platforms, and cancer cell lines (Table S1). We merged 8 studies through an empirical Bayes algorithm [6] to create an internal training and validation set, after reserving 30% of the samples in GSE34228 and GSE10696 for an external validation set with the createDataPartition function from R package Caret. This function performs a stratified random split of the data by sampling within each class to preserve the overall class distribution [7]. These studies were selected because they were the only cohorts with large enough sample sizes for this purpose.

**Figure 2:**
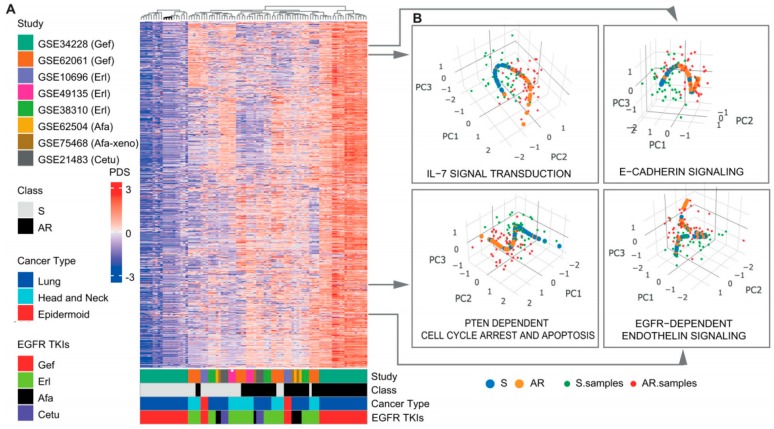
Meta-analysis-derived pathway deregulation analysis. (**A**) Pathway dysregulation score (PDS) matrix for the 8 internal training/validation study sets. Each row represents the z-score-normalized PDS for each individual sample in each cohort. The color-bars in the bottom indicate the following from top to bottom: (1) the study cohort. (2) The resistance status of samples. (3) The cancer subtype of the samples. (4) The type of EGFR-TKI. (**B**) Principal curves of selected pathways. The principal curve learned for the pathways on the 8 study cohort. The data points and the principal curve are projected onto the three principal components (PCs; PC1 to PC3). The principal curve goes through the cloud of samples and is directed so that EGFR-TKI-sensitive samples are near the beginning of the curve. The acquired EGFR-TKI-resistant samples are projected onto the curve. AR, acquired resistance; S, sensitive; Gef, Gefitinib; Erl, Erlotinib; Afa, Afatinib; Cetu, Cetuximab.

**Figure 3:**
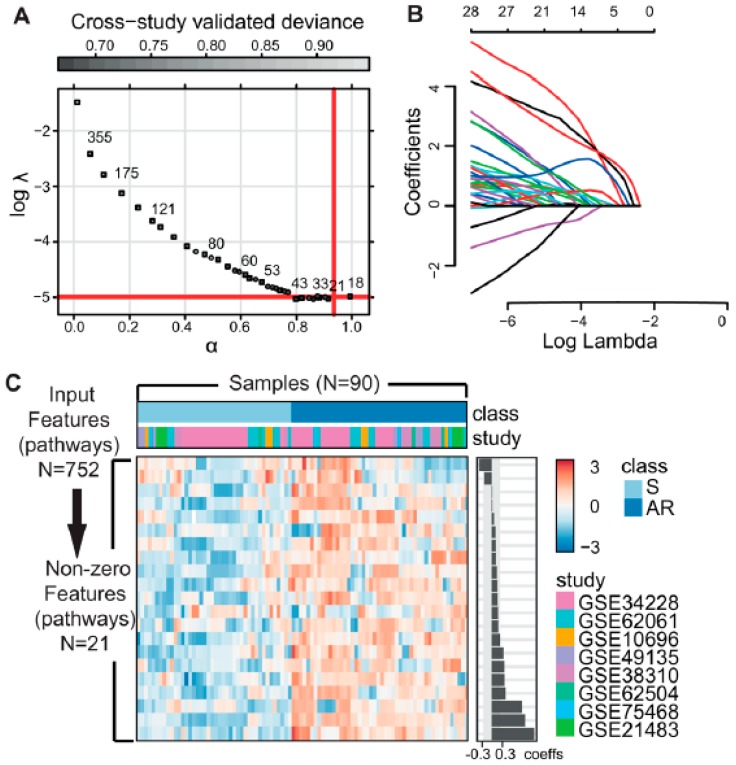
Optimizing meta-analysis-derived elastic net using LOSOCV. (**A**) Hyperparameter optimization for elastic net with EPSGO. Cross validation deviance as a function of both tuning hyperparameters α and log λ. The number of selected features in minimum deviance is shown next to the symbol. The solid lines highlight the final EPSGO solution where the deviance is within 1SE of the minimum. The initial points are plotted as rectangles and iteration points as circles. The optimal parameter values with minimal deviance were found for α = 0.96 and log λ = −4.99, and are highlighted as a solid line. (**B**) Coefficient paths for elastic net penalized regression models applied to the 8 study cohort. The solution path is scaled to reflect log λ on the x-axis. (**C**) Heatmap of the pathways with non-zero coefficients. Sensitive or acquired resistance condition for EGFR-TKIs is indicated above the heatmap. The pathway features are listed in descending order with regard to their coefficient. The optimal hyperparameter values were determined by LOSOCV. AR, acquired resistance; S, sensitive.

**Figure 4:**
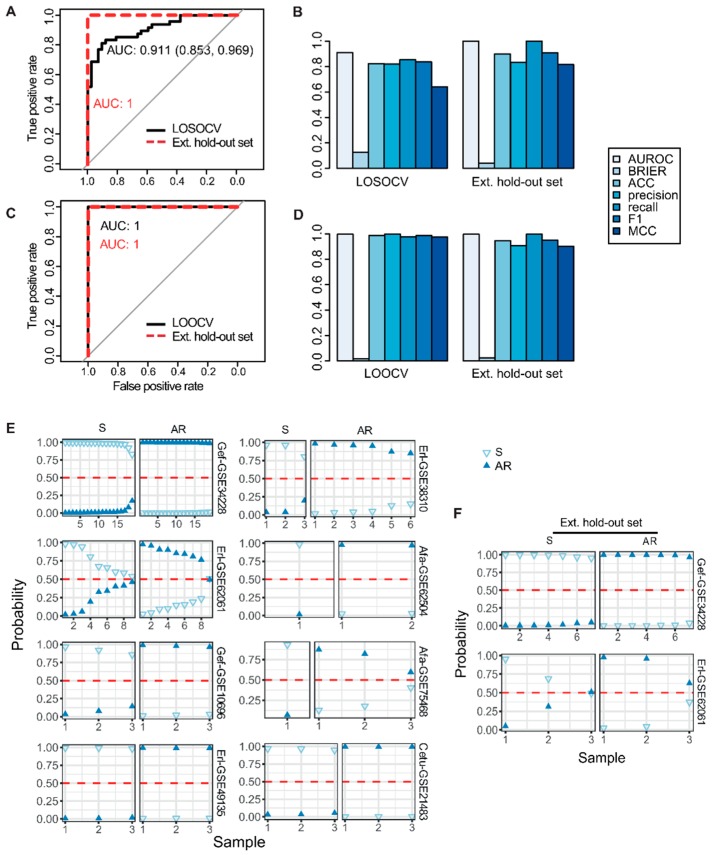
Internal and external evaluation of model performance to distinguish sensitive and acquired resistance to EGFR TKIs. (**A**) Receiver operating characteristic (ROC) curves for the binary classifier in the leave-one-study-out cross validation (LOSOCV). The black line indicates the cross-validation curve, and the dotted red line indicates the external test set. The curve shows sensitivity versus specificity, based on probabilities computed through elastic net regression. (**B**) Different performance metrics (Brier, ACC, precision, recall, F1, and MCC) for the evaluation of classification in LOSOCV. (**C**) Receiver operating characteristic (ROC) curves for the binary classifier in the leave-one-out cross validation (LOOCV). (**D**) Different performance metrics (Brier, ACC, precision, recall, F1, and MCC) for the evaluation of classification in LOOCV. (**E**) Estimated probabilities for samples in cross-study validation. Within study set and subgroup, samples are sorted by the probability of the true group. (**F**) Estimated probabilities for samples in external independent validation. AR, acquired resistance; S, sensitive; ACC, accuracy; MCC, Matthews correlation coefficient.

We then used the Pathifier algorithm to convert the transcriptomics-level data matrix to a pathway-based matrix containing pathway dysregulation scores (PDS) [8]. Recently developed, the Pathifier algorithm is viewed as the best functional class scoring relevant algorithm currently available for deducing pathway level scores. This method finds a principal curve, which nonparametrically and nonlinearly generalizes the first principal component for dimension reduction, using the algorithm by Hastie and Stuetzle [9]. Pathifier produces a one-dimensional principal curve from a cluster of data points in a high-dimensional space. The PDS is a metric that represents the extent of pathway abnormality per sample, and can be calculated using the distance from the starting point of the principal curve to the point projected by a particular individualized pathway. In our study, the initial point was the centroid of the control group, sensitive to EGFR inhibitors. A PDS can range from 0 to 1, with a score closer to 1 indicating a more abnormal pathway. Using this method, it is possible to represent samples using fewer, but more informative variables, based on prior biological pathway knowledge [8]. Applying pathway information from curated databases, including the Kyoto Encyclopedia of Genes and Genomes (KEGG) [10], BioCarta [11], and the National Cancer Institute–Nature Pathway Interaction Database [12], we obtained principal curves for each pathway, and a PDS matrix with 752 rows (pathway features) and 90 columns (samples) (Figure 2A,B). With this PDS matrix, we then used a meta-analysis-based penalized regression method to construct a prediction model for AR to EGFR inhibitors. Penalized regression approaches such as lasso, ridge, and elastic net have been developed to address the challenges caused by high dimensionality of the feature space [4,13,14]. These methods have recently been used to successfully analyze high dimensional human genetic data [4,15,16]. Regression coefficients are shrunk by adding a penalty function to the loss function, which potentially introduces bias but also reduces model variance. Elastic net is a linear combination of lasso and ridge penalties. Two hyperparameters (α and λ) are calibrated for an optimal elastic net penalty function. The α hyperparameter adjusts the levels of contributions from the ridge (L2-norm penalty) and lasso penalties (L1-norm penalty), while λ controls the overall degree of penalization [14]. We used a meta-heuristic algorithm called efficient parameter selection via global optimization (EPSGO) [17], rather than the commonly used fixed grid search methods which are highly arbitrary (see Materials and Methods section for details). Elastic net showed excellent performance on leave-one-out cross-validation (LOOCV), compared to ridge or lasso regression, and EPSGO-tuned elastic net further increased the discrimination power of the classifier (Figures S1 and S2, Table S2). Consequently, EPSGO tuning was employed to find the optimal values of α and λ with minimum binomial deviance (Figure 3A). These optimal parameter values were used for feature selection (Figure 3B,C, Figure S2). At the value for which the penalization parameter gave the lowest cross validation error, the overall area under curve of receiver operating characteristic (AUROC) of the classifier was 0.91 and 1 for the LOSOCV and LOOCV settings, respectively (Figure 4A,C and Figure S3). The results were quite surprising, because all eight studies in the cohort came from different types of cancer cell lines, EGFR inhibitors, and technology platforms (Figure 2A and Table S1). This suggests that pathway-based features have high transferability and generalizability. In addition, other performance metrics (F1, precision, recall, Brier score, accuracy, and Matthews correlation coefficient (MCC)) that examine prediction error further support the predictive power of this model (Figure 4B,D, Figure S3 and Table S5).

The leave-one-study-out strategy gave a more parsimonious model with 21 non-zero pathway coefficients, compared to 55 features by the leave-one-out strategy, suggesting that this model is more interpretable and has less risk of overfitting (Figure 3A and Figure S2C). The detailed results are given in Tables S3 and S4. Next, we further validated our model using an independent blind test set (Gef-GSE34228 and Erl-GSE10696) that was not used in model discovery. The resulting pathway-based predictive model still achieved very high performance on the independent test sets, with perfect AUCs of 1 for both the Gef and Erl sets (Figure 4A,C). Moreover, the additional evaluation metrics also confirmed the robustness and generality of our meta-analysis-based pathway-based learning model (Figure 4B,D and Table S5).

## 3. Discussion

Most EGFR inhibitor resistance predictive models use genomic predictors such as gene signatures, filtered with arbitrary cutoff values and often hard to interpret. The use of a meta-analytic approach and pathway features offers a more robust and comprehensive look into underlying biological processes than individual genes. The novelty and strength of our approach is that we considered all dimensions and applied pathway mapping to a multi-study model to build a generalized predictive model for AR to EGFR inhibitors. Through this, we achieved excellent predictive performance for both the cross study validation set and the independent blind test set.

Our study employed a two-step approach to dimensionality reduction: cross-study pathway-level representation and penalized regression with a global-tuning algorithm. The first step of complexity reduction is to convert individual gene-level information into pathway-level information. A growing body of evidence suggests that pathway-based features can provide more insight into the biological aspects of disease prediction [8,18]. In our study, although the cohort was highly heterogeneous, the model performed remarkably well, which suggests that pathway-based features are good representatives of the true phenotypes. The second step of complexity reduction is regularization. Due to the intrinsic nature of high dimensionality, the low sample size, and heterogeneity of the studies we employed, a regularized regression approach was paired with a fine-tuning algorithm to build a generalized classifier for EGFR inhibitor resistance. This regularization regression is comprised of a loss function with a penalty function, with the latter function placing a heavier penalty on more complex models. The severity of the penalty is tuned empirically using cross-study validation in addition to the more traditional cross validation approach, and is then further optimized using the state-of-the-art EPSGO algorithm to find the global optimization parameter. This process provides additional reduction in model complexity and increases model interpretability.

From the 752 pathways used for the analysis, LOSOCV selected 21 non-zero pathway coefficients for the final model, reflecting much more sparsity than the final model by LOOCV, which contains 55 non-zero features (Table S3). The common genes shared in more than 10 pathways were PI3K, AKT1, MAPK1, SRC, SHC1, FYN, and GRB2. All of them are known to play a central role in EGFR-mediated signaling pathways (Table S4 and Figure S4B). The majority of the pathways are closely related to previously identified potential EGFR inhibitor drug resistance pathways (NCI’s ‘Regulation of p38-alpha and p38-beta’ [19]; NCI’s ‘E−Cadherin signaling’ pathway’ [19]; ‘Hedgehog signaling events mediated by Gli proteins’ [20]; ‘Atypical NF-kb pathway’ [21]; BioCarta’s ‘PTEN dependent cell cycle arrest and apoptosis’ [22]; ‘CXCR4 signaling pathway’ [23]; ‘Hypoxia-inducible factor in the cardiovascular system’ [23]). The associations between the rest of the pathways and acquired resistance are relatively unexplored and require follow-up functional studies. One of them is BioCarta’s ER associated degradation (ERAD) pathway, which had the highest non-zero coefficient (Table S3). Traditionally, EGFR proteins are known as cell surface receptors activated by ligand binding, which results in tyrosine kinase activation and downstream signaling. These downstream signaling pathways are crucial for aggressiveness and resistance development of cancers. Recent evidence has indicated that EGFR receptors are transported from the cell surface to the nucleus, and transmit signals to influence a variety of biological functions. It has been hypothesized that EGFR receptors are shuttled to the cytoplasm through the ERAD pathway, and to the nucleus through the nuclear pore complex (NPC) and importin-β [24]. Nuclear EGFR has been reported in various tumors, and was associated with poor outcomes [25,26]. One previous study indicated nuclear EGFR is accountable for cetuximab acquired resistance [27]. Further investigation into the ERAD pathway and nuclear EGFR is urgently needed, as it may provide invaluable knowledge into acquired resistance. Some of the others are directly involved in growth factor signaling, among them the NCI’s ‘EGFR-dependent Endothelin signaling events’ and ‘Ephrin a reverse signaling pathway’. Nectins and DeltaNp63 signaling pathways are known to be implicated in the tumor progression and anticancer drug resistance [28,29], but their potential roles in EGFR inhibitors resistance have not yet been studied. Three out of 21 non-zero pathways are metabolic pathways. Two of them are associated with the biosynthesis of fatty acids, and the other with phenylalanine metabolism (Table S3 and Figure S4A). Glycosylated sphingolipids are involved in the formation of lipid rafts, which have long been suggested to play an important role in the development of multidrug resistance (MDR) [30]. It has been reported that EGFR is commonly localized to lipid rafts, most prominently in the EGFR TKI resistant cell lines [31]. Phenylalanine has been shown to have the potential to suppress the MDR phenotype [32]. However, whether phenylalanine metabolism is involved in EGFR TKI resistance had not been reported. A better understanding of these pathway features could potentially serve as a basis for discovering the mechanism of resistance development.

Having parsimony and transferability without losing predictive capacity is very important in models, especially for medical applications. This is the first study of its kind to report such high validation accuracy and transferability over different types of cancer cell lines and EGFR inhibitors. In this study, using a state-of-the art machine learning technique, we successfully developed a meta-analysis-derived, multivariate model for personalized pathways in acquired EGFR inhibitor resistance that is able to accurately identify general predictors.

## 4. Materials and Methods

### 4.1. Data Set Configurations

Eight publicly available study cohorts (GSE34228, GSE10696, GSE62061, GSE49135, GSE38310, GSE62504, GSE75468, GSE21483) [33,34,35,36,37,38] only included samples that were stepwise selected for acquired resistant cell lines and encompassed 4 different types of EGFR inhibitors (gefitinib, erlotinib, afatinib and cetuximab), 3 types of cancer (lung, head and neck, and epidermoid cancer), and 4 types of array platforms (Table S1). GSE75468 included acquired afatinib-resistant non-small cell lung cancer cell lines derived from a tumor xenograft model. We excluded studies with insufficient information on the type of drug resistance (innate or acquired). Animal studies and studies with extremely small sample sizes or an inadequate control conditions were also ruled out. The selection process resulted in a total of eight studies to be included in the study cohort. Of these, the gefininb (GSE34228) and erlotinib (GSE62061) studies had large enough sample sizes to be partially used to construct an external test set. Stratified random sampling was used to select 30% of the samples from each study for external use. The other six studies were solely used for model training and cross-study validation due to the smaller sample sizes. Detailed information of the study subjects is given in Table S1.

### 4.2. Data Processing

All data used in this paper is publicly available from the Gene Expression Omnibus (GEO). Normalization and log-transformation of expression values from each dataset were performed as previously described in detail [15]. If raw data from Affymetrix platforms were available, they were pre-processed by robust multi-array average (RMA) [15]. Otherwise, we used pre-processed data from the authors. For gene level summarization, we employed an interquartile range (IQR) method, in which we selected the probe set ID with the largest IQR of expression values among all multiple probe set IDs to represent the gene. Cross-study normalization to correct batch effect was performed using the ComBat function in the sva R package [39]. ComBat uses an empirical Bayes method, which tunes data to remove batch effects and is very effective for datasets with small sample sizes [6]. Blind sets for external validation were not used in internal cross-study normalization to prevent any effects in model building, which established the model’s generalizability to predict from any unknown data [15]. In external validation, we used ComBat for cross-study normalization for each addition of a blind set using the same protocol. Next, as biological pathways are the aggregate of gene activities and generally much more robust than gene markers, we converted gene-wise information to pathway-wise information to detect the common features for acquired drug resistance, regardless of EGFR inhibitors and cancer cell lines [40,41].

### 4.3. Pathway Mapping

Pathway dysregulation scores (PDS) for each individual sample were calculated using a pipeline that employed the Pathifier algorithm as previously described [7]. Pathifier is a non-linear method for quantifying degree of pathway abnormality. The algorithm learns the standard pathway flow from control samples and utilizes this to construct a principal curve. Every sample is projected onto this principal curve, and the PDS is calculated from the normalized projection distance for each sample’s pathway. Pathway information used to form PDS matrix was extracted from ConsensusPathDB (CPDB) (http://consensuspathdb.org/) [42], which comprises curated information from BioCarta, Kyoto Encyclopedia of Genes and Genomes (KEGG), and the National Cancer Institute—Nature Pathway Interaction Database. We used the R package pathifier [8] to calculate PDS.

### 4.4. Model Building

We built the prediction model using elastic net regularization using the R package glmnet [13]. Friedman et al. [13,14] describe the elastic net algorithm in detail. To construct the meta-analysis-derived classifier, we referred to and modified the function from R package C060 and a pre-published script by Sill et al. [43], which is available online. We built additional wrapper functions for the glmnet algorithm to fit and tune the model. We used leave-one-study-out cross validation (LOSOCV) and leave-one-out cross validation (LOOCV) to find the optimal value of the regularization parameter with both minimum deviance and minimum deviance + 1SE. In LOSOCV, one study was then taken as the validation set for testing the model, and the remaining studies were used as training data. The cross-validation procedure was repeated for the number of studies to estimate the average standard error and find the optimal parameter values. The efficient parameter selection via global optimization (EPSGO) algorithm was then used to further fine-tune the parameter [17]. EPSGO is a meta-heuristic algorithm which bases its learning an online Gaussian process, and its parameters are chosen by maximum likelihood. Compared to the grid search method, this algorithm is computationally efficient and robust against local minima. LOOCV followed the same process, except for using a sample in place of a study. The optimal parameter values were then used for variable selection.

### 4.5. Evaluation Strategies

We mainly used area under receiver operation characteristic curve (AUROC) to assess the model’s performance. In the context of binary classification, the classifier can produce four possible outcomes: true positive (TP), true negative (TN), false positive (FP), and false negative (FN). The ratio of true positives over the sum of ground truth positives is called the true positive rate (TPR, also known as sensitivity or recall), and is expressed as TP/(TP + FN). The ratio of false positives over the sum of ground truth negatives is called the false positive rate (FPR or 1-specificity), and is expressed as FP/(FP + TN). AUROC is the true positive rate as a function of the false positive rate, and measures the aggregated classification performance with its value ranging between 0.5 and 1. A value of 0.5 corresponds to a random guess, while 1 means a perfect prediction. Precision is the ratio of true positives over the sum of predicted positives, and is expressed as TP/(TP + FP). Precision recall curve summarizes the model performance in terms of precision and recall. F-score is the harmonic mean of precision and recall, expressed as 2*recall*precision/(recall + precision). Brier score is the mean squared error between predicted probabilities and the actual outcome. MCC, taking all four outcomes (TP, TN, FP, and FN) into account and expressed as (TP*TN) − (FP*FN)/square root((TP + FP)*(TP + FN)*(TN + FP)*(TN + FN)), is a geometric mean corrected for chance agreement and generally regarded as a balanced measure. All statistic measures except the Brier score are directly proportional to predictive performance. For the Brier score, higher values denote worse performances. MCC has a range from −1 (completely incorrect) to 1 (completely correct). All other metrics mentioned above have a range of (0, 1). All statistical evaluation and visualization were performed in the R software environment.

## 5. Conclusions

Accurate prediction of chemotherapy resistance is clinically crucial for the management of cancers. Using pathway mapping and machine learning algorithms, we developed a pipeline to build a meta-analysis-based, multivariate model for personalized prediction. Our model achieved high prediction accuracy with generalizability and transferability through robust internal cross-study validation and external validation, enabling personalized prediction for resistance over different types of cancer cell lines and EGFR inhibitors, including gefitinib, erlotinib, afatinib, and cetuximab. From 752 pieces of pathway information, LOSOCV selected 21 pathway coefficients, which was sparser than LOOCV. The highest non-zero coefficient for a pathway was BioCarta’s ER associated degradation (ERAD) pathway, which is implicated in the shuttling of nuclear EGFR into the cytoplasm before its eventual translocation into the nucleus. Further molecular and clinical confirmations are urgently needed, as the associations of nuclear EGFR with various cancers and resistance to cetuximab have been previously described.

## Supplementary Materials

The following are available online at http://www.mdpi.com/2072-6694/11/1/45/s1, Figure S1: Performance comparison of the four classifiers including ridge, lasso, elastic net, and EPSGO-elastic net on the merged cohort. (A) Receiver operating characteristic (ROC) and Precision-Recall curves of four classifiers. (B) Different performance metrics for the evaluation of classification. EPSGO, Efficient Parameter Selection via Global Optimization; AUROC, area under curve of receiver operating characteristic; ACC, accuracy; MCC, Matthews correlation coefficient. Figure S2: Log loss as a function of the regularization hyper-parameter λ for LOSOCV (A) and LOOCV (B) on the merged cohort. Points and, error bars correspond to the mean and the standard deviation, respectively. The dashed lines indicate the final λ solution where the minimum deviance + 1SE was recorded. (C) meta-analysis-derived elastic net with LOOCV. The heatmap shows the pathways with non-zero coefficents. AR, acquired resistance; S, sensitive; LOSOCV, leave-one-study-out cross validation; LOOCV, leave-one-out cross validation. Figure S3: Precision-Recall curves for the binary classifiers ability to distinguish sensitive and acquired resistance to EGFR TKIs in the internal leave-one-study-out (left) or leave-one-sample-out (right) CV (green) and external test set (red). Figure S4: (A) additional principal curves of selected pathways. (B) overlapping gene count in the 752 pathways listed (left) and genes shared in more than 5 pathways (right). Table S1: Characteristics of individual studies. Table S2: The performances of four penalized regression models. Table S3: Pathways with non-zero coefficients using LOOCV and LOSOCV. Table S4: The genes that overlaps between pathways (overlap counts ≥ 3). Table S5: Performance scores for internal and validation.
