# Supplementary material for: Personalized Prediction of Acquired Resistance to EGFR-Targeted Inhibitors Using a Pathway-Based Machine Learning Approach
Source: Cancers (Basel). 2019 Jan 4;11(1):45. doi: 10.3390/cancers11010045 (PMC6357167; doi:10.3390/cancers11010045)
Supplement: Supplementary file 1 [file cancers-11-00045-s001.pdf]

Supplementary Materials: Personalized Prediction of Acquired Resistance to EGFR-Targeted Inhibitors Using a Pathway-Based Machine Learning Approach

Young Rae Kim, Yong Wan Kim, Suh Eun Lee, Hye Won Yang and Sung Young Kim

Table S1. Characteristics of individual studies.

Drug	Dataset	Sample Size		Origin of Cancer (Cell Lines)	Platform
		S	AR		
Gefitinib	GSE34228	26	26	Lung cancer (PC9)	Agilent-014850 Whole Human Genome Microarray 4x44K
	GSE10696	3	3	Epidermoid carcinoma (A431)	Affymetrix Human Genome U133 Plus 2.0
Erlotinib	GSE62061	12	12	Head and neck cancer (Cal-27, SSC-25, FaDu, SQ20B)	Illumina HumanHT-12 V4.0 expression beadchip
	GSE49135	3	3	Head and neck cancer (HN5)	Illumina HumanHT-12 V4.0 expression beadchip
	GSE38310	3	6	Lung cancer (HCC827, ER3, T15-2)	Illumina HumanHT-12 V3.0 expression beadchip
Afatinib	GSE62504	1	2	Lung cancer (HCC827)	Illumina HumanHT-12 V3.0 expression beadchip
	GSE75468	1	3	Lung cancer * (HCC827)	Illumina HumanHT-12 V4.0 expression beadchip
Cetuximab	GSE21483	3	3	Head and neck cancer (SCC1)	Affymetrix Human Genome U133 Plus 2.0 Array

GEO, gene expression omnibus; GSE, gene expression series; S, sensitive; AR, acquired EGFR-TKI resistant; * Lung Cancer Cells Derived from Tumor Xenograft Model.

Table S2. The performances of four penalized regression models.

Model	ACC	precision	recall	F1	MCC	AUROC	BRIER
Ridge	0.889	0.852	0.958	0.902	0.782	0.964	0.129
Lasso	0.944	0.957	0.938	0.947	0.889	0.991	0.042
Elastic Net	0.978	0.979	0.979	0.979	0.955	0.999	0.023
EPSGO Elastic Net	0.989	1.000	0.979	0.989	0.978	1.000	0.018

AUROC, area under curve of receiver operating characteristic; ACC, accuracy; MCC, Matthews correlation coefficient; EPSGO, Efficient Parameter Selection via Global Optimization algorithm.

Table S3. Pathways with non-zero coefficients using LOOCV and LOSOCV.

No.	Pathways with nonzero coefficients (*n* =55)	Non-zero coefficients	Source	Pathways with non-zero coefficients using LOSOCV (*n* = 21)
1	REGULATION OF P38-ALPHA AND P38-BETA	−1.766503612	PID	TRUE
2	PROTEOGLYCAN SYNDECAN-MEDIATED SIGNALING EVENTS	−1.136281573	PID	TRUE

3	EPHRIN B REVERSE SIGNALING	−0.721317963	PID	FALSE
4	CELL TO CELL ADHESION SIGNALING	−0.477917154	BioCarta	FALSE
5	ERK1-ERK2 MAPK SIGNALING PATHWAY	−0.199265242	BioCarta	FALSE
6	INTEGRIN SIGNALING PATHWAY	−0.128014066	BioCarta	FALSE
7	HEDGEHOG SIGNALING PATHWAY	−0.005571133	KEGG	FALSE
8	TELOMERES TELOMERASE CELLULAR AGING AND IMMORTALITY	0.011199268	BioCarta	FALSE
9	ROLE OF EGF RECEPTOR TRANSACTIVATION BY GPCRS IN CARDIAC HYPERTROPHY	0.024523643	BioCarta	FALSE
10	DOWNREGULATED OF MTA-3 IN ER-NEGATIVE BREAST TUMORS	0.053349923	BioCarta	FALSE
11	FGF SIGNALING PATHWAY	0.075131747	PID	FALSE
12	MECHANISM OF PROTEIN IMPORT INTO THE NUCLEUS	0.08164103	BioCarta	FALSE
13	ROLE OF PI3K SUBUNIT P85 IN REGULATION OF ACTIN ORGANIZATION AND CELL MIGRATION	0.117438736	BioCarta	FALSE
14	IL2 SIGNALING EVENTS MEDIATED BY PI3K	0.168333374	PID	FALSE
15	ROLE OF NICOTINIC ACETYLCHOLINE RECEPTORS IN THE REGULATION OF APOPTOSIS	0.174536482	BioCarta	FALSE
16	NITROGEN METABOLISM	0.183164709	KEGG	FALSE
17	ONE CARBON POOL BY FOLATE	0.195622076	KEGG	FALSE
18	EPHA2 FORWARD SIGNALING	0.20421591	PID	FALSE
19	S1P5 PATHWAY	0.211230972	PID	FALSE
20	G-PROTEIN SIGNALING THROUGH TUBBY PROTEINS	0.216111841	BioCarta	FALSE
21	CDK REGULATION OF DNA REPLICATION	0.264061143	BioCarta	FALSE
22	CYCLING OF RAN IN NUCLEOCYTOPLASMIC TRANSPORT	0.283930054	BioCarta	FALSE
23	NEPHRIN-NEPH1 SIGNALING IN THE KIDNEY PODOCYTE	0.298814913	PID	FALSE
24	HYPOXIA-INDUCIBLE FACTOR IN THE CARDIVASCULAR SYSTEM	0.303271487	BioCarta	TRUE
25	EPHA FORWARD SIGNALING	0.313490344	PID	FALSE
26	SYNDECAN-1-MEDIATED SIGNALING EVENTS	0.318816363	PID	FALSE
27	GALACTOSE METABOLISM	0.322466451	KEGG	FALSE
28	WNT SIGNALING NETWORK	0.327314283	PID	FALSE
29	SIGNALING EVENTS MEDIATED BY THE HEDGEHOG FAMILY	0.370292759	PID	FALSE
30	OLFACTORY TRANSDUCTION	0.432674384	KEGG	FALSE
31	NECTIN ADHESION PATHWAY	0.437835458	PID	TRUE
32	ATYPICAL NF-KAPPAB PATHWAY	0.446905522	PID	TRUE
33	REGULATION OF ANDROGEN RECEPTOR ACTIVITY	0.517690779	PID	FALSE
34	ERBB RECEPTOR SIGNALING NETWORK	0.548526238	PID	FALSE
35	CXCR4 SIGNALING PATHWAY	0.562974028	BioCarta	TRUE
36	E-CADHERIN SIGNALING IN KERATINOCYTES	0.64708616	PID	TRUE
37	HEDGEHOG SIGNALING EVENTS MEDIATED BY GLI PROTEINS	0.662081488	PID	TRUE

38	PRION DISEASES	0.676095874	KEGG	FALSE
39	ASPIRIN BLOCKS SIGNALING PATHWAY INVOLVED IN PLATELET ACTIVATION	0.79155655	BioCarta	FALSE
40	THE IGF-1 RECEPTOR AND LONGEVITY	0.805583453	BioCarta	FALSE
41	PHENYLALANINE METABOLISM	0.827221124	KEGG	TRUE
42	IL2-MEDIATED SIGNALING EVENTS	0.920700196	PID	FALSE
43	GLYCOSPHINGOLIPID BIOSYNTHESIS - GANGLIO SERIES	0.997329934	KEGG	TRUE
44	BIOSYNTHESIS OF UNSATURATED FATTY ACIDS	1.059629055	KEGG	TRUE
45	EPHRIN A REVERSE SIGNALING	1.060213506	PID	TRUE
46	CARDIAC PROTECTION AGAINST ROS	1.111659385	BioCarta	FALSE
47	HOW DOES SALMONELLA HIJACK A CELL	1.118940525	BioCarta	TRUE
48	IL-7 SIGNAL TRANSDUCTION	1.155986994	BioCarta	TRUE
49	PROXIMAL TUBULE BICARBONATE RECLAMATION	1.350203916	KEGG	TRUE
50	EGFR-DEPENDENT ENDOTHELIN SIGNALING EVENTS	1.382471164	PID	TRUE
51	PTEN DEPENDENT CELL CYCLE ARREST AND APOPTOSIS	1.417434559	BioCarta	TRUE
52	PHOSPHOLIPASE C DELTA IN PHOSPHOLIPID ASSOCIATED CELL SIGNALING	1.576989062	BioCarta	TRUE
53	VALIDATED TRANSCRIPTIONAL TARGETS OF DELTANP63 ISOFORMS	1.884449116	PID	TRUE
54	ERYTHROPOIETIN MEDIATED NEUROPROTECTION THROUGH NF-KB	2.560218675	BioCarta	TRUE
55	ER ASSOCIATED DEGRADATION -ERAD- PATHWAY	2.79315365	BioCart	TRUE

LOOCV, leave-one-out cross validation; LOSOCV, leave-one-study-out cross validation; Coeffs, coefficients.

Table S4. The genes that overlap between pathways (overlap counts ≥ 3).

No.	**Entrezid**	**HUGO Gene Symbol**	**Genename**	**Overlap Counts**
1	5290	*PIK3CA*	phosphatidylinositol-4,5-bisphosphate 3-kinase, catalytic subunit alpha	16
2	5295	*PIK3R1*	phosphoinositide-3-kinase, regulatory subunit 1 (alpha)	16
3	6714	*SRC*	SRC proto-oncogene, non-receptor tyrosine kinase	13
4	2885	*GRB2*	growth factor receptor-bound protein 2	11
5	207	*AKT1*	v-akt murine thymoma viral oncogene homolog 1	10
6	2534	*FYN*	FYN proto-oncogene, Src family tyrosine kinase	10
7	6464	*SHC1*	SHC (Src homology 2 domain containing) transforming protein 1	10
8	5879	*RAC1*	ras-related C3 botulinum toxin substrate 1 (rho family, small GTP binding protein Rac1)	9
9	6654	*SOS1*	son of sevenless homolog 1 (Drosophila)	9
10	3265	*HRAS*	Harvey rat sarcoma viral oncogene homolog	8
11	387	*RHOA*	ras homolog family member A	8
12	3725	*JUN*	jun proto-oncogene	7
13	3932	*LCK*	LCK proto-oncogene, Src family tyrosine kinase	7
14	5594	*MAPK1*	mitogen-activated protein kinase 1	7

15	5595	*MAPK3*	mitogen-activated protein kinase 3	7
16	5335	*PLCG1*	phospholipase C, gamma 1	6
17	5604	*MAP2K1*	mitogen-activated protein kinase kinase 1	6
18	5747	*PTK2*	protein tyrosine kinase 2	6
19	1432	*MAPK14*	mitogen-activated protein kinase 14	5
20	2782	*GNB1*	guanine nucleotide binding protein (G protein), beta polypeptide 1	5
21	409	*ARRB2*	arrestin, beta 2	5
22	4609	*MYC*	v-myc avian myelocytomatosis viral oncogene homolog	5
23	5566	*PRKACA*	protein kinase, cAMP-dependent, catalytic, alpha	5
24	5599	*MAPK8*	mitogen-activated protein kinase 8	5
25	1906	*EDN1*	endothelin 1	4
26	1956	*EGFR*	epidermal growth factor receptor	4
27	2033	*EP300*	E1A binding protein p300	4
28	2185	*PTK2B*	protein tyrosine kinase 2 beta	4
29	2309	*FOXO3*	forkhead box O3	4
30	2770	*GNAI1*	guanine nucleotide binding protein (G protein), alpha inhibiting activity polypeptide 1	4
31	2792	*GNGT1*	guanine nucleotide binding protein (G protein), gamma transducing activity polypeptide 1	4
32	2932	*GSK3B*	glycogen synthase kinase 3 beta	4
33	3320	*HSP90AA1*	heat shock protein 90kDa alpha (cytosolic), class A member 1	4
34	5605	*MAP2K2*	mitogen-activated protein kinase kinase 2	4
35	5781	*PTPN11*	protein tyrosine phosphatase, non-receptor type 11	4
36	5894	*RAF1*	Raf-1 proto-oncogene, serine/threonine kinase	4
37	596	*BCL2*	B-cell CLL/lymphoma 2	4
38	5970	*RELA*	v-rel avian reticuloendotheliosis viral oncogene homolog A	4
39	6416	*MAP2K4*	mitogen-activated protein kinase kinase 4	4
40	7015	*TERT*	telomerase reverse transcriptase	4
41	9564	*BCAR1*	breast cancer anti-estrogen resistance 1	4
42	998	*CDC42*	cell division cycle 42	4
43	999	*CDH1*	cadherin 1, type 1	4
44	100129518	*LOC100129518*	uncharacterized LOC100129518	3
45	1387	*CREBBP*	CREB binding protein	3
46	142685	*ASB15*	ankyrin repeat and SOCS box containing 15	3
47	1950	*EGF*	epidermal growth factor	3
48	2268	*FGR*	FGR proto-oncogene, Src family tyrosine kinase	3
49	2353	*FOS*	FBJ murine osteosarcoma viral oncogene homolog	3
50	2736	*GLI2*	GLI family zinc finger 2	3
51	3055	*HCK*	HCK proto-oncogene, Src family tyrosine kinase	3
52	3065	*HDAC1*	histone deacetylase 1	3
53	3561	*IL2RG*	interleukin 2 receptor, gamma	3
54	3716	*JAK1*	Janus kinase 1	3
55	3718	*JAK3*	Janus kinase 3	3

56	4067	*LYN*	LYN proto-oncogene, Src family tyrosine kinase	3
57	4792	*NFKBIA*	nuclear factor of kappa light polypeptide gene enhancer in B-cells inhibitor, alpha	3
58	5058	*PAK1*	p21 protein (Cdc42/Rac)-activated kinase 1	3
59	5170	*PDPK1*	3-phosphoinositide dependent protein kinase 1	3
60	5567	*PRKACB*	protein kinase, cAMP-dependent, catalytic, beta	3
61	5568	*PRKACG*	protein kinase, cAMP-dependent, catalytic, gamma	3
62	5578	*PRKCA*	protein kinase C, alpha	3
63	5579	*PRKCB*	protein kinase C, beta	3
64	5613	*PRKX*	protein kinase, X-linked	3
65	5727	*PTCH1*	patched 1	3
66	6195	*RPS6KA1*	ribosomal protein S6 kinase, 90kDa, polypeptide 1	3
67	640	*BLK*	BLK proto-oncogene, Src family tyrosine kinase	3
68	6469	*SHH*	sonic hedgehog	3
69	6608	*SMO*	smoothened, frizzled class receptor	3
70	6647	*SOD1*	superoxide dismutase 1, soluble	3
71	6648	*SOD2*	superoxide dismutase 2, mitochondrial	3
72	6774	*STAT3*	signal transducer and activator of transcription 3 (acute-phase response factor)	3
73	6777	*STAT5B*	signal transducer and activator of transcription 5B	3
74	7410	*VAV2*	vav 2 guanine nucleotide exchange factor	3
75	7525	*YES1*	YES proto-oncogene 1, Src family tyrosine kinase	3
76	8945	*BTRC*	beta-transducin repeat containing E3 ubiquitin protein ligase	3
77	8976	*WASL*	Wiskott-Aldrich syndrome-like	3

Table S5. Performance scores for internal and external validation.

Internal and External Validation	**AUROC**	**BRIER**	**ACC**	**Precision**	**Recall**	**F1**	**MCC**
LOSOCV							
Internal 8 studies cross-study validation	0.911	0.127	0.822	0.820	0.854	0.837	0.642
External validation (GSE34228)	1.000	0.005	1.000	1.000	1.000	1.000	1.000
External validation (GSE62061)	1.000	0.123	0.667	0.600	1.000	0.750	0.447
External validation (overall)	1.000	0.040	0.900	0.833	1.000	0.909	0.861
LOOCV							
Internal 8 studies leave-one-sample-out cross validation	1.000	0.018	0.989	1.000	0.979	0.989	0.978
External validation (GSE34228)	1.000	0.000	1.000	1.000	1.000	1.000	1.000
External validation (GSE62061)	1.000	0.083	0.833	0.750	1.000	0.857	0.707
External validation (overall)	1.000	0.025	0.950	0.909	1.000	0.952	0.905

LOSOCV, leave-one-study-out cross validation; LOOCV, leave-one-out cross validation; AUROC, area under curve of receiver operating characteristic; ACC, accuracy; MCC, Matthews correlation coefficient.

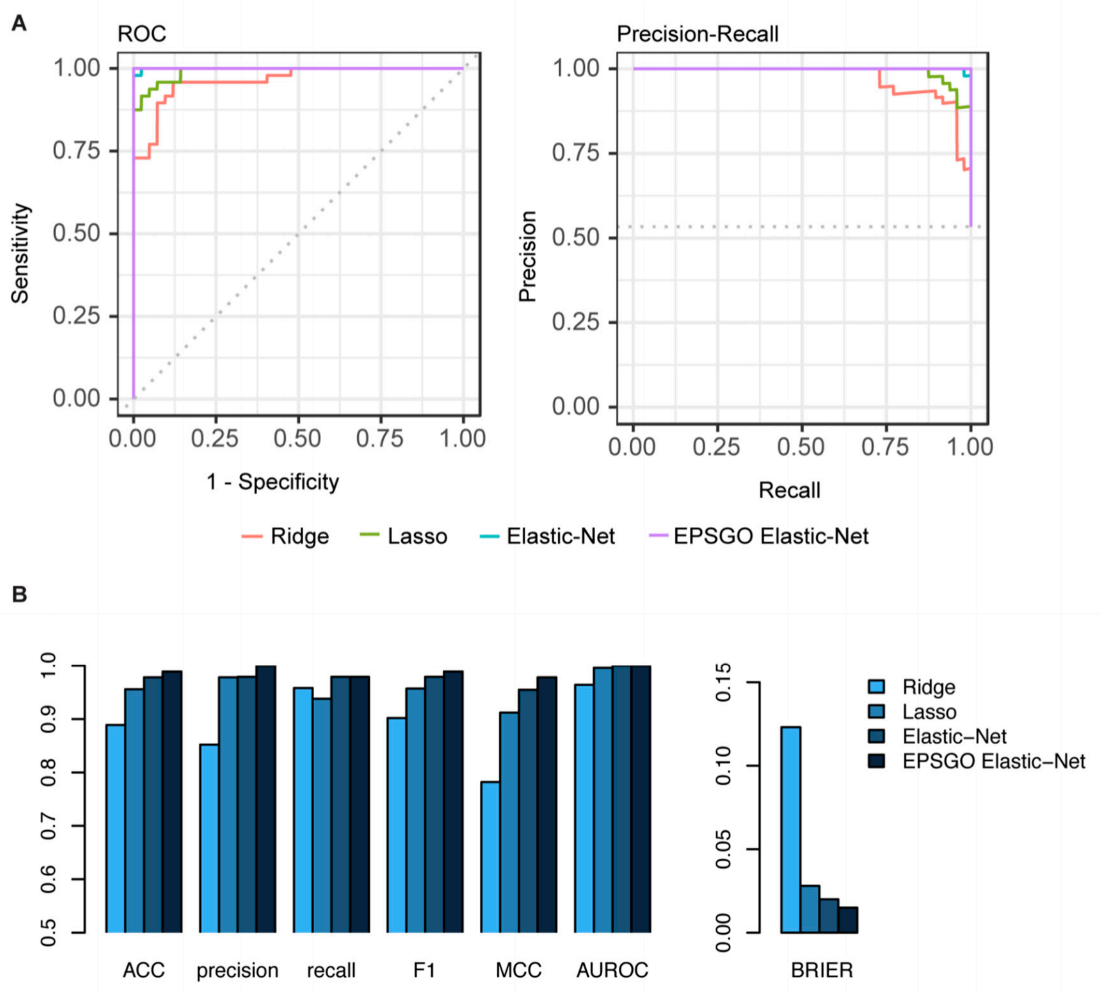

Figure S1. Performance comparison of the four classifiers including ridge, lasso, elastic net and EPSGO-Elastic net on the merged cohort. (**A**), Receiver operating characteristic (ROC) and Precision-Recall curves of four classifiers. (**B**), Different performance metrics for the evaluation of classification. EPSGO, Efficient Parameter Selection via Global Optimization; AUROC, area under curve of receiver operating characteristic; ACC, accuracy; MCC, Matthews correlation coefficient.

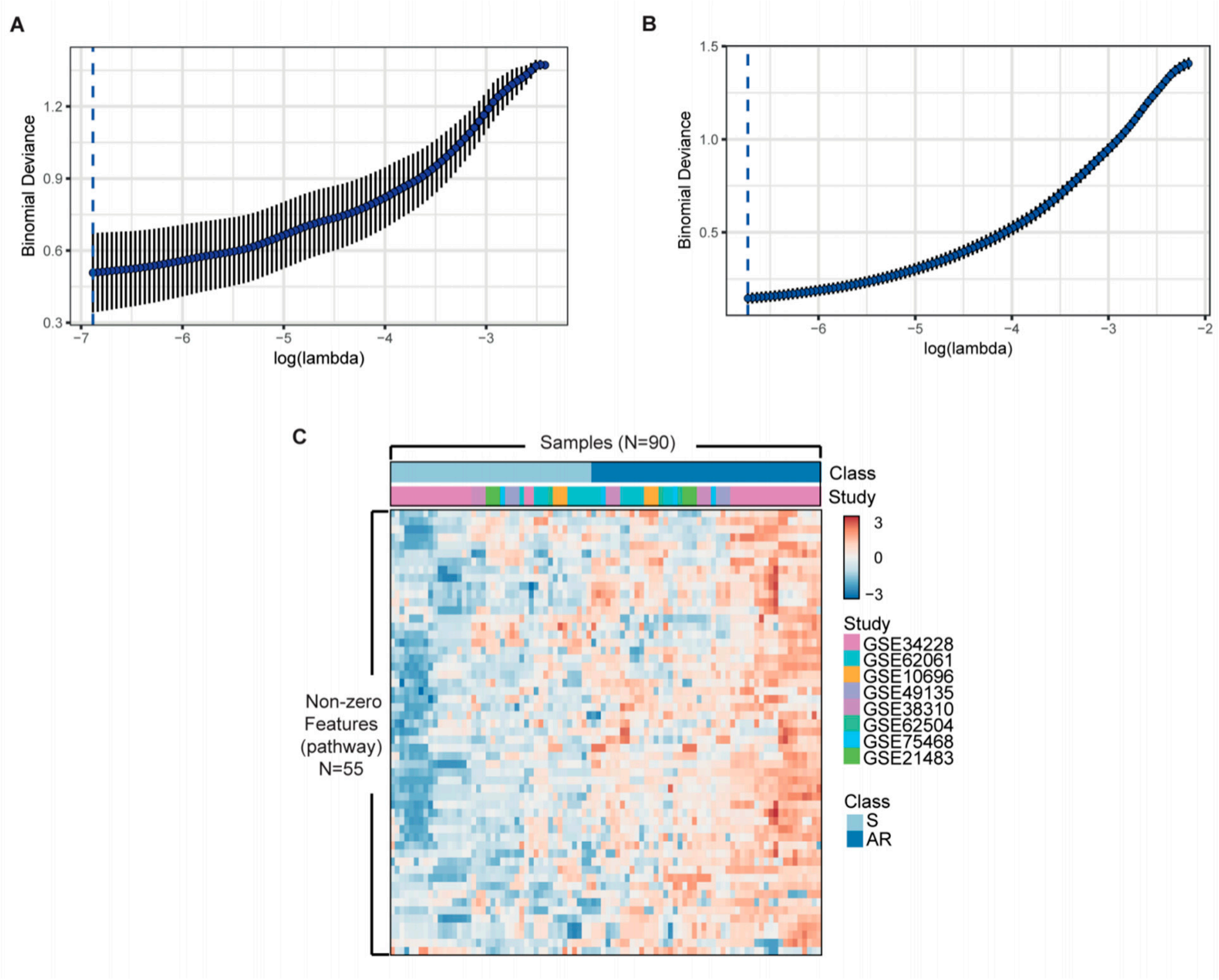

Figure S**2.** Log loss as a function of the regularization hyper-parameter λ for LOSOCV (**A**) and LOOCV (**B**) on the merged cohort. Points and, error bars correspond to he mean and the standard deviation respectively. The dashed lines indicate the final λ solution where the minimum deviance + 1SE was recorded. (**C**), meta-analysis-derived Elastic Net with LOOCV. The heatmap shows the pathways with non-zero coefficent. AR, acquired resistance; S, sensitive; LOSOCV, leave-one-study-out cross validation; LOOCV, leave-one-out cross validation.

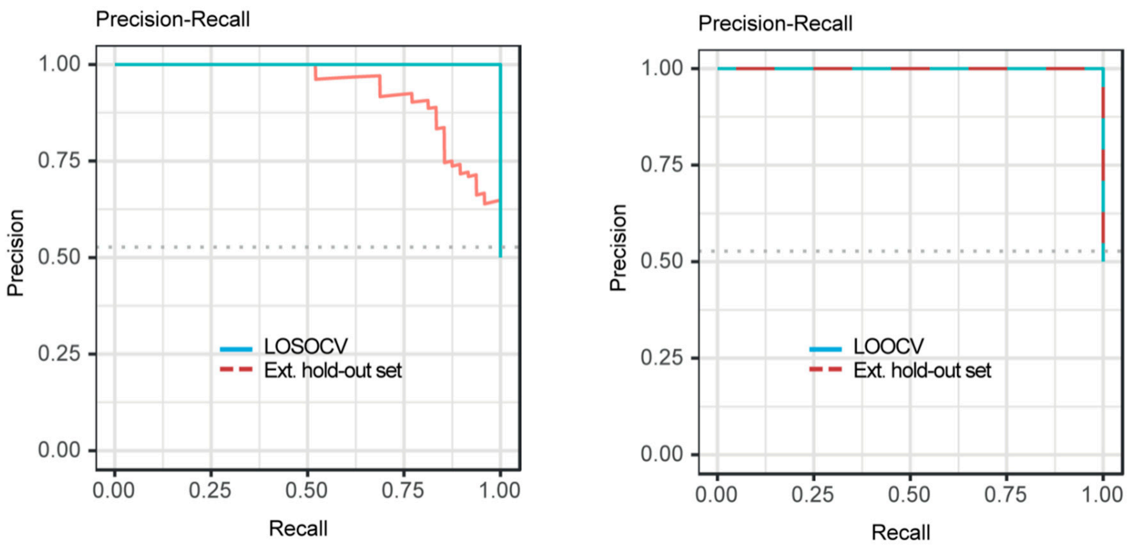

Figure S3. Precision-Recall curves for the binary classifiers ability to distinguish sensitive and acquired resistance to EGFR TKIs in the internal leave-one-study-out (left) or leave-one-sample-out (right) CV (green) and external test set (red).

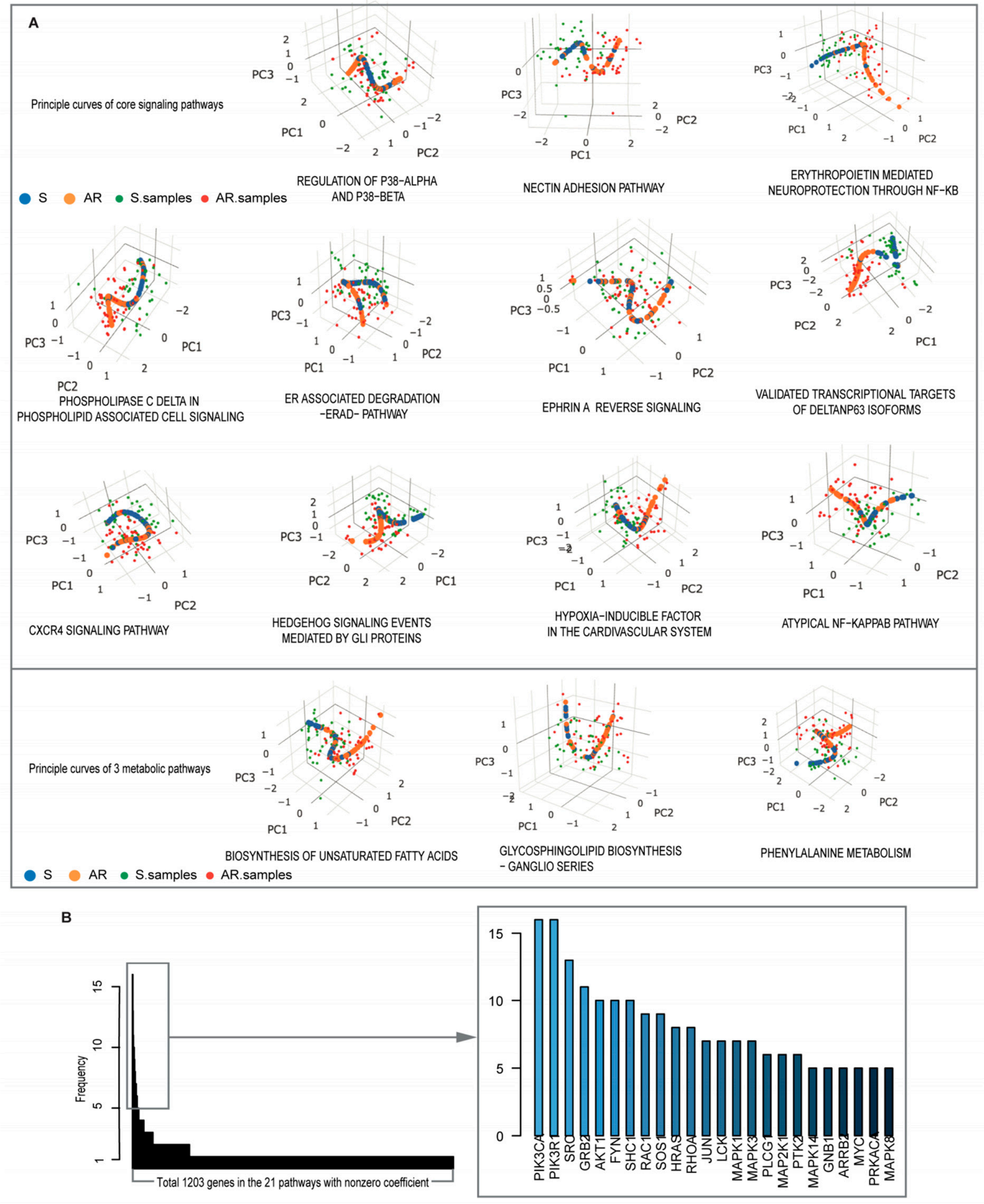

Figure S4. (**A**), additional principal curves of selected pathways. (**B**), overlapping gene count in the 752 pathways listed (left) and genes shared in more than 5 pathways (right).

© 2018 by the authors. Licensee MDPI, Basel, Switzerland. This article is an open access article distributed under the terms and conditions of the Creative Commons Attribution (CC BY) license (http://creativecommons.org/licenses/by/4.0/).
